# The new normal for children’s physical activity and screen viewing: a multi-perspective qualitative analysis of behaviours a year after the COVID-19 lockdowns in the UK

**DOI:** 10.1186/s12889-023-16021-y

**Published:** 2023-07-27

**Authors:** Robert Walker, Danielle House, Ruth Salway, Lydia Emm-Collison, Lara E. Hollander, Kate Sansum, Katie Breheny, Sarah Churchward, Joanna G. Williams, Frank de Vocht, William Hollingworth, Charlie Foster, Russell Jago

**Affiliations:** 1 Centre for Exercise, Nutrition & Health Sciences, School for Policy Studies, University of Bristol, Bristol, BS8 ITZ UK; 2 Population Health Sciences, Bristol Medical School, University of Bristol, Bristol, BS8 2PS UK; 3 Independent Public Member of the Project Team, Bristol, UK; 4 Communities and Public Health, Bristol City Council, Bristol, BS1 9NE UK; 5 The National Institute for Health Research, Applied Research Collaboration West (NIHR ARC West), University Hospitals Bristol and Weston NHS Foundation Trust, Bristol, BS1 2NT UK; 6 NIHR Bristol Biomedical Research Centre, University Hospitals Bristol and Weston NHS Foundation Trust and University of Bristol, Bristol, UK

**Keywords:** Clubs, Play, Socioeconomic position, Gender, Mental health, Skills

## Abstract

**Background:**

The COVID-19 pandemic significantly impacted children’s physical activity. Recent evidence indicated children’s accelerometer-measured physical activity levels have, on average, returned to near pre-pandemic levels in 2022, though sedentary behaviour remains higher. However, insufficient physical activity levels among children continues to be a critical public health issue in the UK, with only 41% meeting physical activity guidelines. This study aimed to provide in-depth analysis of how the pandemic has shaped children’s physical activity patterns beyond the short-term periods following lockdowns and identify the new challenges to engaging children in physical activity.

**Methods:**

One-to-one interviews with parents (*n* = 22), school staff (*n* = 9), and six focus groups with children aged 10–11 years (*n* = 45) were conducted between February and July 2022. Topics explored changes to children’s physical activity and sedentary behaviour patterns, including screen-viewing, and factors influencing any changes. The framework method was used for analysis.

**Results:**

Five themes were generated. Theme 1 described residual lockdown habits, including increased screen-viewing within the home, while activities outside the home continued to feel less spontaneous. Theme 2 highlighted an interrupted development of social, emotional, and physical skills among children compared to what would be expected pre-pandemic. This coincided with Theme 3 which reflected increased mental health challenges among families, creating complex barriers to children’s physical activity. A new normal for child physical activity was evoked and explored in Theme 4, with greater dependence on structured and organised activities. However, Theme 5 highlighted that girls and children with lower socio-economic position may be especially at risk of decreased physical activity.

**Conclusions:**

There is a new normal for children’s physical activity that is characterised by increased dependence on structured and organised physical activities, such as active clubs, and less on unstructured and spontaneous physical activities, such as physical play. While this may suit many children, girls and children from lower socio-economic households face barriers to participating in the new normal. It is important that affordable and equitable opportunities are provided to all children to prevent physical activity and health inequalities.

**Supplementary Information:**

The online version contains supplementary material available at 10.1186/s12889-023-16021-y.

## Background

Physical activity has been associated with a number of health and quality of life benefits among children, including decreased cardiometabolic risk factors, risk of depression, and increased academic performance, executive functioning, and attention [1–4]. The World Health Organisation and UK Chief Medical Officers recommend that children should accumulate an average of an hour of moderate-to-vigorous intensity physical activity (MVPA) per day [5–7]. However, large proportions of children do not engage in sufficient levels of physical activity [8–11], with 59% of children aged 10–11 years in the UK estimated to not have been meeting recommended activity levels prior to the COVID-19 pandemic [8].

**Fig. 1:**
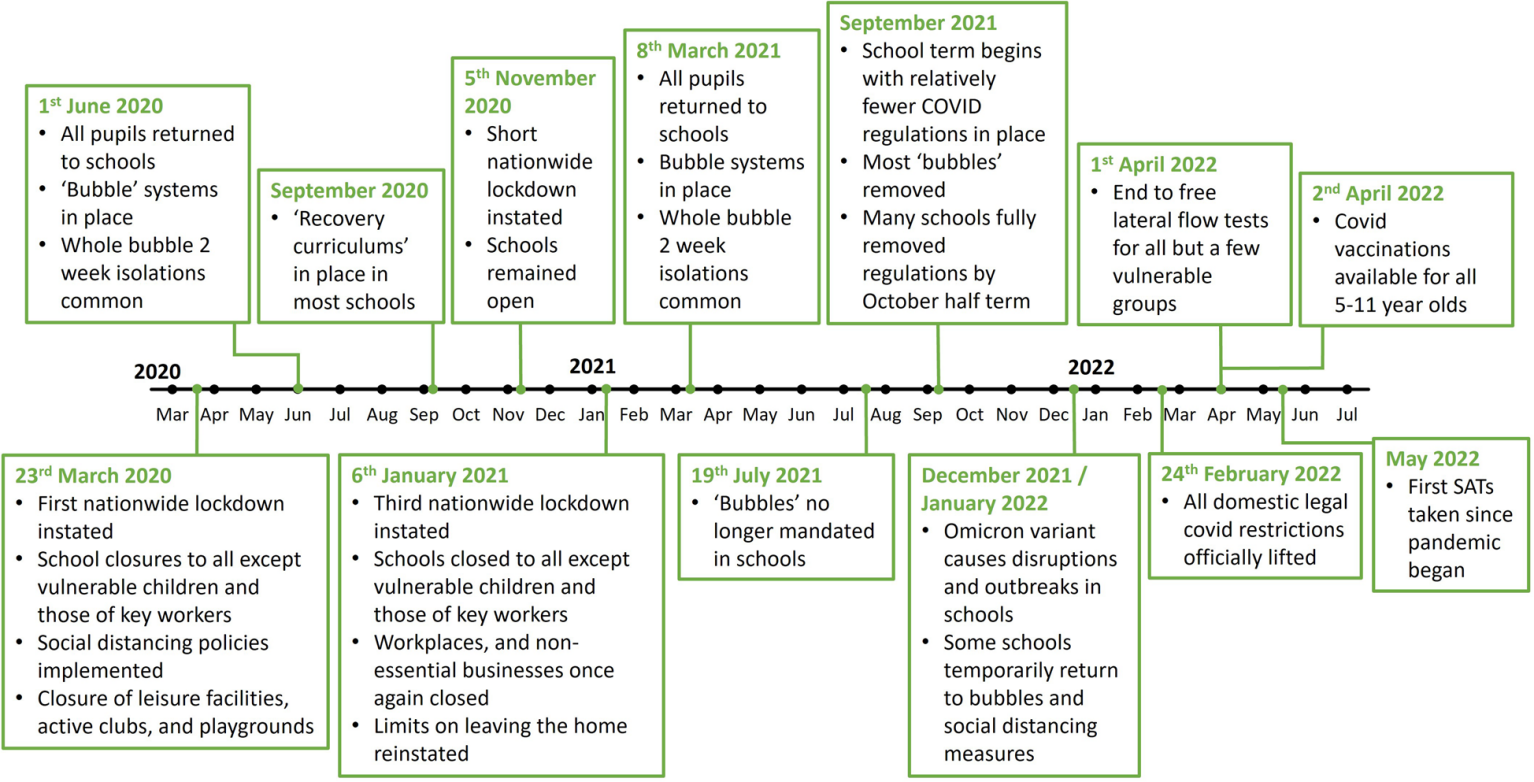
Timeline of COVID-19 restrictions in England

In response to the pandemic, many countries implemented strict lockdowns and restrictions, impacting movement patterns and behaviours globally. A growing body of international research suggests that these responses have negatively impacted children’s physical activity [12–14]. In England, beginning in March 2020, a series of lockdowns, school closures, and other restrictions were implemented to mitigate the spread of COVID-19. The final lockdown was lifted in March 2021 where legal restrictions were gradually eased until fully removed in February 2022. A timeline for England’s COVID-19 response can be seen in Fig. 1.

Active-6 is a multidisciplinary project exploring the impact of the pandemic on children aged 10–11 years and their parents’ physical activity in the UK [15–19]. Analyses of accelerometer data collected between May and December 2021 revealed that children taking part had on average 7–8 fewer minutes of MVPA and 25 additional minutes of sedentary time per day in comparison with a pre-COVID group of similarly aged children from the same schools [16]. These findings are echoed in international research that highlight a negative short-term impact of the pandemic on child physical activity [12–14]. Qualitative research with Active-6 participants and school staff in the latter half of 2021 suggested that children experienced physical fatigue and emotional overwhelm during their return to a lifestyle that more closely reflected a pre-pandemic normality, creating significant challenges to physical activity [17]. Other UK-based qualitative research exploring this topic using the framework method suggested that the closures of schools, active clubs, and other areas of society due to COVID-19 restrictions impacted children’s motivation for physical activity via their feelings of autonomy, competence, and relatedness [20].

Total leisure screen-viewing among Active-6 participants, a sedentary behaviour, was higher post-lockdown compared to pre-pandemic by 11% and 8% on weekdays and weekends respectively, equating to 12–15 min [18]. Television (TV)-viewing was particularly higher by 68% on weekdays and 80% on weekends [18]. Many aspects of parents’ and children’s lives transitioned to screen-based activities during lockdowns, resulting in an unavoidable increase in screen-viewing and subsequent habitualisation that continued post-lockdown [18]. These findings were consistent with the wider international narrative that indicates TV and screen-viewing among primary school-aged children significantly increased during strict lockdowns [21]. It is important to note here that the association between sedentary behaviours, which includes screen-viewing, and physical activity among children is small [22] and that these should not be considered functional opposites, as it is possible to record high levels of both.

Additional accelerometer data was collected from children and parents in 2022 that suggested children’s MVPA had returned to pre-pandemic levels, but sedentary time still remained higher by around 13 min per day [19]. Although these findings imply a stabilisation of children’s physical activity, it is important to note that insufficient levels of physical activity among children continues to be a critical public health issue, with only 41% of children in the UK meeting physical activity guidelines [19].

The recovery in children’s MVPA and elevated sedentary behaviour in 2022 hints at a change to children’s physical activity patterns. To date, Active-6 [17] and other UK-based qualitative research [20] has focused on short-term changes occurring either during lockdowns or in periods immediately after, whilst under other restrictions. It is therefore unclear how the pandemic has shaped children’s activity patterns, including the types of activities in which they engage, beyond periods of COVID-19 restrictions, and whether new challenges have emerged to promoting children’s physical activity in our post-pandemic society. This study aimed to provide in-depth qualitative analysis of these issues beyond periods of legally enforced COVID-19 restrictions, drawing on a unique combination of multiple perspectives.

## Methods

### Participants and procedure

Detailed information regarding the Active-6 project has been published elsewhere [16–19]. In short, the Active-6 project is a repeated cross-sectional natural experiment examining the impact of the COVID-19 pandemic on the accelerometer-measured physical activity and sedentary behaviour of 10–11 year old children (primary school Year 6 in the UK school system) and their parents in the Greater Bristol area, Southwest England. Data for this project were collected in two separate waves, the first in 2021 and second in 2022. Qualitative research was also conducted with parents, children, and school staff who participated in the first or second wave of Active-6. This study draws on qualitative data collected from participants who were involved in the second wave. Sample size was guided by information power [23], whereby the study’s aim, the extent of participants’ specific knowledge and experiences related to the research question, theoretical background of the study, the quality of dialogue, and the adopted cross-case analysis were reflected on and discussed within the research team throughout the data collection process. Demographic information can be seen in Table 1.

**Table 1:** Characteristics of Active-6 interview and focus group participants by demographic, physical activity and job role

	N
**Parents**	**22**
Gender	
Male	7
Female	15
Parent education	
Higher degree	4
Degree	16
A level	2
IMD decile	
≤ 5	5
> 5	17
Parent activity levels	
High MVPA	12
Medium MVPA	7
Low MVPA	3
**School staff**	**9**
Gender	
Male	5
Female	4
Role	
Year 6 teacher	5
Full-time PE Coordinator	2
Headteacher	2
School postcode IMD	
≤ 5	2
> 5	7
**Children**	**45**
Gender	
Male	22
Female	23
Child activity levels	
High MVPA	11
Medium MVPA	17
Low MVPA	17
Home postcode IMD	
≤ 5	13
> 5	32

*IMD decile* ≤ 5 = greater level of deprivation, > 5 = lesser level of deprivation

One-to-one semi-structured interviews were conducted with parents (*n* = 22 from 12 different schools) who had participated in the second wave of the Active-6 project, had worn accelerometers as part of this project, and had consented to being recontacted. Using their accelerometer data, parents were categorised as either low, medium or high MVPA based on their weekday MVPA in comparison to other parents from their school. Although we aimed to recruit a diverse sample, challenges in recruiting male, less active, and lower socio-economic households meant that convenience sampling was used. Household postcodes that were provided during the sign-up process were used to determine Index of Multiple Deprivation (IMD; [24]). IMD ranges from 1–10, with higher IMD score indicating a lower level of deprivation. Parent interviews were conducted by RW between February and July 2022 and ranged from 30 to 71 min (M = 51.4).

Six focus groups were conducted with 45 children from six schools. Focus group size ranged from 5–8 children. Children were also categorised as low, medium, or high MVPA levels and publicly available postcodes of schools were used to determine school area IMD. This information was then used to purposively sample and recruit children with even ratios of gender and low/medium/high MVPA in each group from schools situated in an equal split of urban/rural and high/low deprivation areas via emailing their parents. These children had no relation to this study’s parent interviewees. All focus groups were conducted in-person at the children’s schools between May and June 2022 by RW, DH, and KS and ranged from 43 to 56 min (M = 47.66).

One-to-one semi-structured interviews with school staff (*n* = 9 from 8 schools) who had supported the Active-6 project were directly contacted and invited to participate. Due to the pressured primary school environment, many school staff were unable to give their time to participate. Thus, we adopted a convenience sampling approach. School staff interviews were conducted by RW between May and July 2022 and ranged from 48 to 54 min (M = 51.14).

Interviews and focus groups were recorded using an encrypted Dictaphone. As recompense for their time, parents and school staff were provided with a £10 gift voucher and children had received a small gift for participating in the Active-6 project.

### Study materials

Interview/focus group topic guides focused upon changes to physical activity behaviour among parents and children following January 2022. These guides were informed by the socioecological model [25], with questions included to capture policy, community, organisational, interpersonal, and individual factors. Specific questions were also included based upon findings of a previous Active-6 qualitative study conducted in 2021 [17]. Separate topic guides were developed for each participant group. Parents were asked whether their own and their child’s physical activity and screen-viewing patterns had changed in 2022, and any factors that had influenced these changes. School contact interviews explored changes to, and influences of, activity levels among Year 6 pupils from the school-perspective. The child focus group topic guide was developed to encourage discussions surrounding perceptions of, and changes to, children’s physical activity and screen viewing, and factors influencing any changes. Topic guides can be seen in the supplementary materials.

### Qualitative data analysis

The framework method was used to organise and analyse data [26]. This method was selected as the summarised frameworks helped to reduce the large quantities of qualitative data associated with this analysis, allowing the multidisciplinary Active-6 team, each with different research experience, to be able to actively participate and engage with the data.

The data analysis process consisted of seven stages: 1) verbatim transcription by a university approved transcription service; 2) data familiarisation; 3) coding; 4) developing a working analytical framework using inductive and deductive codes; 5) applying the analytical framework; 6) charting data into the framework matrix; and 7) interpreting the data. RW, DH, and KS independently coded two transcripts for each participant group using a mixture of inductive and deductive codes. Deductive codes were based upon key themes from a previous qualitative analysis [17] and activity types. Interview content and interpretations were then discussed, and a separate codebook was collaboratively developed for each participant group that RW then applied to the remaining transcripts. Independent coding of transcripts was used to challenge researcher reflexivity and support a deeper and more nuanced analysis. Following this, data was summarised and charted into the framework matrix by LH and reviewed by RW and DH.

This qualitative analysis was based in critical realism [27]. Critical realism is a philosophical meta-theory that posits that a world exists independently of human beings. However, our understanding of this external world is derived through perceptions and descriptions, which is mediated by language, culture, and human practices. This approach allowed rich analysis of multiple perspectives in the context of other quantitative and qualitative findings within the Active-6 project.

## Results

**Fig. 2:**
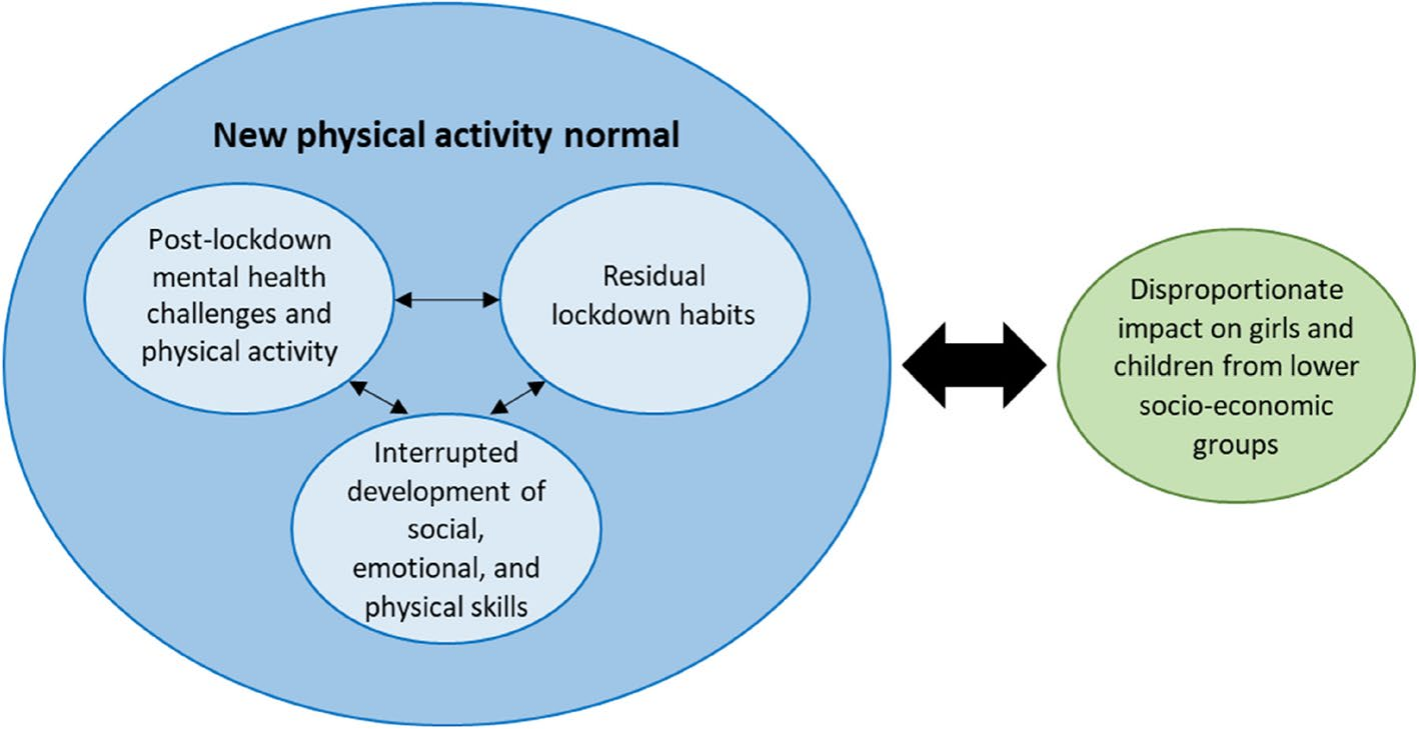
Thematic map with hypothesised relationships between themes

Five themes were generated related to children’s physical activity and screen-viewing a year following the lifting of COVID-19 lockdowns. These were: 1) Residual lockdown habits; 2) Interrupted skill development; 3) Post-lockdown mental health challenges and physical activity; 4) New physical activity normal; and 5) Disproportionate impact on girls and children from lower socio-economic groups. Themes were constructed around a central organising concept and definitions developed as short abstracts to illustrate the scope and boundaries of each theme (Table 2). All five themes were reflected within data across all three participant groups. A thematic map with hypothesised theme relationships can be seen in Fig. 2.

**Table 2:** Theme names and definitions

**Theme name**	**Theme definition**
1) Residual lockdown habits	This theme describes the continuation of habits formed during prolonged periods spent within the home under COVID-19 lockdowns and restrictions. Time spent within the home was suggested to reflect the lifestyle of lockdown, with activities centred around sedentary screen-viewing behaviours. Despite the easing of restrictions, activities outside the home felt intentional and a subsequent reduction in spontaneous physical activity was evoked in the data. Poor diet and sleeping patterns, and their negative effect on physical activity, were also described as habits of lockdown that were apparent a year following the COVID-19 lockdowns.
2) Interrupted skill development	The impact of missed developmental experiences among children due to the lockdowns and restrictions was suggested in the data. Facets of this theme included interrupted social, emotional, and physical skills, each of which created challenges to physical activity.
3) Post-lockdown mental health challenges and physical activity	This theme illustrates a perceived lasting impact of the pandemic on the mental health of parents and children. An increased requirement of mental health support in schools for children and their families was suggested in the data. These challenges to mental health among parents and children were thought to create significant difficulties with regards to being active and encouraged sedentary habits within the home.
4) New physical activity normal	The pandemic was perceived to have had a lasting impact on the activity patterns of children. Due to increased screen-viewing at home, reduced spontaneous activities outside the home, and lasting organisation-level rules and restrictions, the opportunities for spontaneous physical activity have decreased. Subsequently, physical activities that are structured and organised, such as active clubs, were suggested as the primary way in which children are now active.
5) Disproportionate impact on girls and children from lower socio-economic groups	This theme highlights a differential impact among two groups: girls and children with lower socio-economic position. These groups may be at risk of lower physical activity in the wake of the pandemic, despite an average return to pre-pandemic levels within the overall Active-6 study. They may also experience complex, multi-faceted barriers that might create challenges to physical activity, especially attending organised and structured activities, such as active clubs, which have become the new normal of child physical activity.

### Theme 1 – Residual lockdown habits

This theme reflects the continuation of habits formed during prolonged periods under lockdowns and restrictions. Despite the lifting of lockdowns in the UK, participants described lasting sedentary behaviours, reminiscent of their lockdown lifestyle. Many parents and school staff thought that children were at a particularly critical age in relation to habit formation, with these younger years being vital to the development of life-long healthy habits. However, due to their age, children might have been less able to understand the importance of behaviours that promoted health during the pandemic, leaving many to be drawn to sedentary behaviours, such as screen-viewing.

*“All of those healthy habits are really entrenched [for adults] where maybe, for the kids, they didn’t have those habits or maybe those habits weren’t intellectualised in such a way… They were happy to just hang out on the sofa for a couple of years and not do anything.” (Parent 8, female, IMD decile 5)*

Screen-viewing activities were a prominent residual habit of the pandemic. Participants evoked a sense of normalisation and persistent default use of electronic devices when at home that often discouraged or reduced the time for other activities. Participants felt that an increased provision of and exposure to screen-based activities meant that children no longer needed to physically leave the home to socialise with friends, reducing opportunities and what was conveyed as a primary motivation for unstructured physical activity among children. Regulating children’s screen-viewing, however, had become increasingly challenging as children aged and gained more independence.

*“I do really like watching it [TV]. I would rather be out doing stuff, but if I have the opportunity to do it, I do just turn to TV...” (Child focus group 3, girl)*

*“…it’s… habit forming… when they get to secondary age and they’ve got more of that independence, I think there is that… assumption that at the end of the school day or of your evenings or your weekend, you’ll be sat home and stuck to a screen rather than maybe going out and doing things because you just haven’t done that for a period of time… you used to have to be forced out of the house to, kind of, have a social life – but now you can put on a headset and all your mates are there… you’re not forced to, kind of, do that physical activity side.” (Parent 1, male, IMD decile 8)*

Yet, changes to habits were not limited to within the home. Though many parents expressed little to no remaining fears and worries related to the COVID-19 virus, the impact of the lockdowns and restrictions on their outdoor movement behaviours was suggested. The strict restrictions during the pandemic reduced family and child independent mobility, with activities at this time needing to be purposeful (i.e., daily exercise, essential shopping). Despite restrictions being lifted, participants noted a lasting feeling that their activities needed to be intentional, reducing spontaneous physical activity.

*“because of the lockdowns, we got ourselves into a routine of, “We go to this place because we have to go there. Then we go straight back home again.” Because it was so rigid... just simple things, like [before the pandemic] after school she might… hang around in the playground and play... All of the little kids would do that, so she did that all the time in the younger years…. Then, of course, lockdown hit… They had to be walked out straightaway to their parents, and then you went home… That’s a small example of physical activity gone…” (Parent 16, female, IMD decile 10)*

Habits formed during lockdowns impacted on many aspects of child health. One headteacher recalled their observations related to children eating poorer quality food and sleeping less, where the mental health challenges associated with living under strict lockdowns and restrictions (Theme 3) encouraged poor eating and sleeping patterns. Subsequently, these behaviours were habitualised over the course of the pandemic, influencing a decline in physical activity.

*“I’ve noticed a significant decline in the quality of food children are having in their packed lunch… I put it down to bad habits learnt over lockdown, just carrying on… a significant portion of children only eat highly processed, highly refined, sugary foods and nothing else…. and big bags of it... That’s quite shocking, in terms of how much that’s changed… It’s easy, cheap food. “I was feeling a bit glum, so we’re going to treat ourselves to that extra chocolate bar,” and it’s just become the norm… getting to sleep and other things seemed to massively deteriorate… if you’re not sleeping, you’re eating rubbish, it’s no wonder your activity levels drop” (School contact 7, School ID 71, headteacher)*

### Theme 2 – Interrupted skill development

This theme illustrates a perceived interrupted development of social, emotional, and physical skills. Opportunities to learn wider skills were lost due to lockdowns and restrictions. A prominent aspect of this theme relates to an interrupted development of social skills, particularly in terms of social confidence and connection with others. A sense of awkwardness and difficulty in reconnecting with peers was expressed by many children, a challenge echoing in their emotions even after a year following the COVID-19 lockdowns. Children felt that this reduced the amount they physically played with friends, a factor that may be encouraging more screen-based play (Theme 1).

*“I wasn’t really able to connect with any of my friends in lockdown. So, when we came back, it was awkward to see everyone… I still feel like I’m a bit awkward and not really sure what’s going on with everyone... I still do activities outside with my friends but not as much as we used to do” (Child focus group 1, boy)*

Lasting social challenges were also suggested to potentially discourage active club attendance, with feelings of social awkwardness leading to avoidance of what they felt could be a difficult and uncomfortable new social situation.

*“I think she lost a little bit of social confidence… she’s a bit of an introvert so to get her joining clubs where she doesn’t know people… is probably a bit of a stretch. She’d rather just give it a miss than feel slightly awkward” (Parent 8, female, IMD decile 5)*

Observations from the school-perspective provided insight related to differences between children who experienced the pandemic and their predecessors. Time spent in isolation, away from their peers, was perceived to have significantly impacted children’s ability to harmoniously interact with one another. Within school-based physical activity, active play and the ability to follow the structure and rules within games were key challenges conveyed throughout the data, often manifesting as social conflict. While some participants felt that social skills were slightly improving following the return to school and subsequent increase in social opportunities, significant persisting challenges in Physical Education (PE) and sport were noted among all age groups of children.

*“There still is [issues surrounding social skills]… Getting children to conform to a set of school rules has been quite tricky... Having to collaborate and work as a team… as soon as it becomes a group there’s lots of conflict. I think sports help provide that conflict resolution practice. It is getting better but I think there’s still a way to go…” (School contact 2, School ID 81, Year 6 teacher)*

Resilience as a crucial emotional skill among children was highlighted by participants and constituted another area where skill development was perceived to be interrupted. A distinct tangible, emotional challenge reengaging with school posed lasting difficulties for some children and teachers. Many teachers felt this originated from a loss of resilience that occurred during the long periods that children spent in the “safety blanket” (School contact 6) of their homes, which negatively impacted many aspects of children’s education, such as the ability to focus on written schoolwork and inability to cope with the physical discomfort brought on by PE.

*“[Some children at the school] don’t appear to be as emotionally developed as children would normally be. They’re not as resilient... They’re certainly not as willing to put any sort of focus into their written work as they normally would do. That, again, translates when we go out to do PE… They come in in the morning and they are either crying or they seem completely disengaged. Then we see children who are not resilient in terms of their own health… [During PE] they might feel a bit hot and they’re like, “Oh, can you ring my mum, I want to go home” whereas normally it would be, “Let’s have a drink of water and we’ll see how we are in half an hour.”… Because they spent a long time at home, that then becomes that safety blanket… We’re still seeing it now...” (School contact 6, School ID 74, dedicated PE coordinator)*

School staff also noted an evident decrease in the physical ability of children that many felt stemmed from prolonged periods in the sedentary lifestyle of lockdown and other restrictions. One headteacher observed significant decreases in a high-achieving school athletics club that continued to win despite the children’s decreased performance, which they suggested reflected a broader nationwide decrease, even among children who are motivated to compete at a high level.

*“We’ve got a track team and it has always been the kind of flagship club at the school… I’ve got data here, probably, six or seven years old. The times are significantly lower for significantly more children than we’re getting now… basically the top performers were running at about 6 min… Whereas you go to the data now… the fastest ones are 7 min… Yet, we’re still winning [competitions]… So, I think this is a drop across the board… So, even those children who were motivated to attend clubs… even their fitness has declined, and their stamina has declined quite significantly…” (School contact 7, School ID 71, headteacher)*

Some parents noticed a loss of physical ability within their child. Swimming was a frequently discussed area that parents observed an interruption of their child’s skills due to limited access to pools that disrupted previously established habits (Theme 1); however, some described a recovery of their child’s swimming ability to near pre-pandemic levels following a return to the pool. While some parents felt that aspects of physical capability had recovered to pre-pandemic levels, others were concerned at a noticeable increase in their child’s body weight that, when combined with a drop in physical ability, created a reluctance towards physical activity.

*“[The swimming pool] was closed for a long time and then once they reopened we managed to go a couple of times at the weekend. It was… a little bit of a shock to the system but they soon got it. After a few weeks they were… swimming probably not far off where they would’ve been otherwise.” (Parent 5, male, IMD decile 6)*

*“I know that they’re probably heavier than the weight that they have been all their lives so maybe that’s impacting on their reluctance to [be physically active]… If you lose a bit of fitness you’re kind of a bit sludgy for a while, aren’t you, until you get moving again. Maybe they just have never moved enough to get over that sludgy feeling.” (Parent 8, female, IMD decile 5)*

The impact of reduced physical ability was also apparent among children who discussed a persisting sense of fatigue and tiredness, discouraging activities such as child independent mobility and physical play, while encouraging sedentary behaviours after school (Theme 1).

*“Before COVID and all that, I’d be outside pretty much all day but after that I’d only be outside 30 min and I have to come in… I don’t know [why that is]… I get a lot tired for some reason…” (Child focus group 6, boy)*

### Theme 3 – Post-lockdown mental health challenges and physical activity

This theme describes the perceived increase in child and parent mental health challenges as a legacy of the pandemic, and its impact on children’s physical activity. One member of school staff labelled this phase of the pandemic the “post-traumatic period” (School contact 1). A mixture of exhaustion and a need to process the experiences of the last few years created feelings for pupils that contrasted with what they felt was expected within the current societal narrative of a return to pre-pandemic “normality”.

*“I think it possibly is improving but I think that, in terms of [pupil’s] mental health, the effects of being at home for a long time and the unknowingness of being in school, the changes in school… I just think it [the pandemic] takes a lot of time for any human to process… Certainly a lot of head teachers that I’ve spoken to have said, “This [academic year 2021-2022] has been the hardest year.”… we are in a post-traumatic time now… it’s the hardest time because you’ve got that fatigue from the last two years… We’re aware that it doesn’t feel back to normal but we’re being told it’s back to normal...” (School contact 1, School ID 44, headteacher)*

These lasting mental health challenges were evidenced in the experiences of some school staff who observed an increased need for mental health support among some children and their families. While some participants felt that these issues were improving, others feared they were getting worse.

*“For me, the most striking thing is that there are increasingly more children … not just the children… Families and possibly older siblings that are requiring supporting from mental health services...” (School contact 5, School ID 64, Year 6 teacher)*

Participants felt that parents or children who were experiencing mental health challenges faced significant barriers to children’s physical activity. For example, encouraging children to leave the home could require significant effort by the parent when children were uncooperative, which some parents expressed was considerably more difficult when their mental health was low. This theme was suggested to be reciprocal with children’s and parent’s physical activity and sedentary habits within the home (Theme 1), where mental health issues made returning to activities outside the home more difficult but subsequently negatively impacted mental health.

*“I can think of several parents at school where their mental health is quite depleted and they just can’t face getting their boots on, their coats on… sometimes just getting the kids out the front door is not just a case of: ‘Get your coat on. We’re going out.’…” (Parent 3, female, IMD decile 8)*

### Theme 4 – New physical activity normal

The fourth theme illustrates the idea of a new physical activity and screen-viewing normal formed over the pandemic. Time spent within the home has become more centred around screen-based activities, whilst much of the time spent outside of the home consists of intentional activities (Theme 1). Subsequently, opportunities for spontaneous physical activity were perceived to have decreased, replaced by organised physical activity scheduled at a specific time slot within the week.

*“…everything becomes allotted to a time slot... That whole, kind of, concept of building things into your day, into your week or the… flexibility of it or the ad hoc… nature of play… if you don’t create an opportunity for it, it won’t happen. Whereas, I think it used to be something that you would be running, you would be doing different things… There’s not so much of that there.” (Parent 1, male, IMD decile 8)*

The transition to more structured and organised physical activities was not only attributable to habitualised behaviours but exacerbated by lasting societal changes and rules surrounding entering facilities. The continuation of booking systems that were implemented during the pandemic in a range of activities, such as heritage sites and swimming, despite legal restrictions being lifted were noted by participants. Restrictions on the number of people that can enter a facility at one time were seen to make booking activities competitive, with a need to schedule in advance. This caused additional barriers and frustrations for families who found it difficult to take advantage of good weather and unanticipated free time. Considering the impact of facility closures and restrictions on the swimming ability of children and the importance of returning to the pool (Theme 2), these additional barriers could significantly impact the long-term swimming ability of many children.

*“…something I find, now, frustrating after the pandemic... you have to book tickets to go to things like family days out… Like National Trust things… you have to book a timeslot… I appreciate they’re doing [it] to limit numbers but I’m quite a spontaneous person so you kind of want to wait for the weather… If the tickets are always booked, you can’t… Previously you could just turn up on the day… I think some of that spontaneity has probably gone.” (Parent 10*, *female, IMD decile 9)*

In this new normal, active clubs were described as a prominent structured activity and remedy to the habitualised screen-based, sedentary lifestyle. The necessity of an organised period where children would intentionally leave the home and attend a structured activity was suggested as an important and effective method for ensuring children were active. Many parents expressed a need for multiple club attendance, which created time where children are able to be active away from screens and counteract the increased sedentary lifestyle at home.

*“…he’s got a games console. He’s got a phone, he’s got an access to a TV… he would just rotate between the three all day, if he could. He doesn’t seem to have a desire to, kind of, move away from those…. if something’s programmed in, planned in, then he would, you know, he knows that he’s going to go and play football, he’s going to go and play rugby, but he hasn’t got that desire to even move away from [electronic devices] and go into the kitchen… go to the garden and do, sort of, activity himself.” (Parent 1, male, IMD decile 8)*

### Theme 5 – Disproportionate impact on girls and children from lower socio-economic groups

Despite evidence that suggests activity levels among children have returned to pre-pandemic levels [19], this qualitative analysis highlights that some sub-groups may now be at risk of lower physical activity.

The pandemic has shaped the norms of physical activity among children, with greater dependence on organised activities, such as active clubs (Theme 4). However, these activities can be costly, and combined with increased cost of living challenges in the UK over the past year, fee paying activities incur costs that some families could not justify.

*“In general kids’ extra-curricular clubs are expensive… It’s why we haven’t put our boys into swimming lessons, because it’s too much. We can’t afford it… at the moment with the cost of living, which we’ve become really aware… I know people who have dropped gymnastics, cheerleading...” (Parent 15, female, IMD decile 2)*

Yet, financial barriers appeared to be a complex issue. For example, one member of school staff explained that despite the removal of financial barriers for children with lower socio-economic position, these children still did not attend, suggesting that barriers may not be limited to the financial domain. As time spent within the home has become characterised by screen-viewing among many families (Theme 1), this may lead to an increased sedentary behaviour among children with lower socio-economic position.

*“…there’re always some children who don’t [participate in school active clubs]… Especially free school meals or pupil premium children… don’t tend to [participate]… Even though we don’t charge...” (School contact 4, School ID 71, Year 6 teacher)*

The impact of the pandemic on girls’ physical activity was also highlighted in the data, with a perceived increase in reluctance among girls to participate in certain forms of physical activity, such as some active clubs. This was reflected in the experiences of one parent who expressed concern for their daughter’s recent loss of interest in an active club they had previously enjoyed, despite parental encouragement.

*“She definitely dropped out of her dance programme because of COVID, she never went back… she really loved it, she made friends through that, that she still has, but neither of them went back to dance… We really tried to encourage her to pick that up again and she just wasn’t interested.” (Parent 8, female, IMD decile 5)*

School staff felt that girls’ perception of sport and physical activity had changed over the course of the pandemic, stemming from a lack of exposure during the pandemic. One Year 6 class teacher described that girls are particularly vulnerable to such breaks as they start to become more self and body-aware earlier than boys, which leads to avoidance of activities that they feel may cause embarrassment. Considering the increased dependence on active clubs in the new normal of physical activity (Theme 4), this could create significant differences between girls’ and boys’ levels of physical activity.

*“In my particular class, a lot of girls are not pro joining clubs… Anything that is external and competitive, a lot of the girls in my class shy away from… I wonder whether because they might have had maybe an 18-month, 2-year gap of competing… I think maybe that embarrassment or self-awareness maybe kicks in a little bit earlier for girls. Because they haven’t sat with it for two years, to go back to it feels maybe a bit alien...” (School contact 9, School ID 61, Year 6 teacher)*

## Discussion

Drawing on the perspectives of children, parents, and school staff, this study adds narrative and understanding related to the physical activity and screen-viewing behaviour of children in the UK a year following the COVID-19 lockdowns.

A key finding from our analysis is that habits formed during lockdowns have continued a year later. The development of habitual behaviours is reliant on the presence of contextual cues [28]. For example, attending an active club at a certain time and place each week leads to regular physical activity that may become habitual. Responses to mitigate the spread of COVID-19 brought significant changes to children’s daily environment, with many spending an increased amount of time within the home due to school and active club closures, and restrictions on movement. Researchers have suggested that fluctuations in physical activity among children over the pandemic may be explained by prolonged periods of restrictions interrupting cues and habits associated with physical activity, that required time to re-learn once opportunities became available [14, 19]. Our qualitative analysis provides nuance and detail to the role of habits in explaining changes to child physical activity. Namely, habits were not only influential to physical activity through active behaviours, such as child independent mobility, active play, and active club participation, but also as an interrelated system of screen-viewing, diet, and sleep behaviours. Established screen-viewing behaviours were a prominent residual habit of lockdown, as also reflected in a related mixed methods study [18] and wider literature [21, 29]. Time spent at home during lockdowns may have established cues within the home, leading to automatic inclinations towards screen-viewing behaviours. Our analysis further indicates that the known age-related increases in child screen-viewing [30, 31] may be starting earlier.

The impact of the pandemic on mental health and wellbeing has been identified as an international issue [32–34]. Although physical activity for the promotion and protection of children’s current and future mental health is a well-researched area [3, 35], the impact of child and parent mental health barriers to child physical activity are less known. Coupled with what participants expressed as an interrupted development of emotional, social, and physical skills, children may face unique challenges to physical activity post-pandemic. Subsequently, context-specific school-based interventions that consider these emerging challenges offer a promising means of developing children’s skills and increasing physical activity in the wake of the pandemic [36].

Research has shown that children’s physical activity transitions from informal physical activity, such as active play, during younger years to more formal forms of physical activity, such as active clubs, as the child becomes older [37, 38]. Our qualitative analysis suggests that these age-related declines in unstructured physical activity may be occurring faster than pre-pandemic. These changes have been occurring for some time as we move towards reduced active travel [39], reduced independent mobility and unstructured play [40–43] and subsequently more dependent on physical activity that is scheduled and adult-led [44]. Despite this accelerated shift towards structured forms of physical activity post-pandemic, it is important that unstructured physical activity continues to be promoted so that children who do not have access or are less inclined to participate in structured forms of physical activity have the opportunity to be active. In addition, understanding the social drivers for these changes in children’s activity patterns and how we can promote a mixture of structured and unstructured physical activity across all periods of the day is likely to be essential for the future health of the nation. Thus, future research is warranted to see how types of children’s physical activity differ pre- and post-lockdown so that differences in activity patterns as well as overall physical activity levels can be better understood.

Although MVPA levels among children have generally returned to pre-pandemic levels [19, 45], it is important to highlight groups that may not have recovered. Health inequalities among lower socio-economic groups is a prominent public health issue in the UK, with 35.6% of premature deaths attributable to socio-economic inequality [46]. A disproportionate number of these deaths were caused by obesity [46] which has been shown to relate to lower individual and area socio-economic position among children [47]. The WHO cites reduced levels of obesity (adiposity) among children as a critical outcome of meeting physical activity guidelines [6]. However, unlike obesity, research reveals a complex, inconsistent picture of socio-economic position and child physical activity, with some pre-pandemic studies identifying a relationship between socio-economic position and child physical activity [48], while others identified few or no differences [37, 47]. Lower socio-economic position during childhood has been linked with less leisure-time physical activity behaviour in adulthood [49, 50], and was cited as a constraining factor during the pandemic [12, 17], with physical activity among children and young people going to school in the most deprived area in UK not yet recovering to pre-pandemic levels [45]. Research conducted in the USA suggested that the home environment of groups with lower socio-economic position facilitate sedentary behaviour rather than physical activity, with children having more access to media, such as TVs, in their bedrooms while having less access to portable play equipment, such as bikes [51]. Children from lower socio-economic groups have also been shown to watch more TV with their family members than those from higher-socio-economic groups [51]. Our results build upon this, suggesting that organised activities such as active clubs, the new normal of child physical activity, may be less accessible to children with lower socio-economic position, particularly with the increasing rise in cost of living in the UK [52], which subsequently may make them more likely to be drawn to screen-viewing habits within the home. This provides possible explanation to the lack of recovery among children and young people attending school in the most deprived areas of the UK [45]. Future research is needed to further explore and promote physical activity in this group.

Boys participate in more physical activity than girls [37, 45]. The impact of the lockdowns and restrictions on child physical activity also differed by gender, with physical activity among boys negatively affected more than girls [12, 19, 53]. Subsequently, boys’ physical activity increased and recovered to near pre-pandemic levels following the removal of restrictions, whereas girls’ physical activity remained relatively stable throughout, re-establishing typical pre-pandemic gender differences post-lockdown [19, 53]. Our analysis suggests that the pandemic may have exacerbated pre-existing gender barriers to physical activity among girls, such as extended interruptions and lack of exposure to physical activity due to lockdowns influencing girls’ perceptions of physical activity. As a result, girls may be less likely to engage in organised physical activities, such as active clubs, the new physical activity normal for children. Thus, there is a need to explore these issues further and provide ways to help more girls, and perhaps especially girls from lower socio-economic groups, to be physically active.

### Study implications

Key findings and implications from this study can be seen in Table 3. The primary message from this study is the new normal of physical activity, where children have become more dependent on organised physical activity, that has coincided with changes in physical activity and sedentary habits, mental health challenges and interrupted development of skills. Although the new physical activity normal may suit many children, girls and children from lower socio-economic groups may be at particular risk of lower than average levels of physical activity.

**Table 3:** Key findings and implications

**Key finding**	**Implications**
Disproportionate impact of the pandemic on the physical activity behaviour among girls and children from lower socio-economic groups	Girls and children from lower socio-economic groups may be at a greater risk of lower physical activity levels. Practitioners would benefit from considering the unique barriers and challenges within these groups in order to ensure the equitable promotion of health and wellbeing
Interrupted development of social, emotional, and physical skills	Strategies to promote physical, social, and emotional development opportunities for children are warranted. Practitioners may benefit from drawing on sport and other physical activities to promote development opportunities for children
Increased dependence on active clubs for physical activity	Ensuring that sufficient, affordable, and accessible active club provision is available for all children is important for encouraging physical activity. Exploring the barriers and challenges to increasing provision is warranted to optimise provision

### Strengths and limitations

This study had several strengths. The interrelated narrative of the Active-6 project allowed this study to draw upon context-specific mixed-method results to add depth to this analysis. Inclusion of observations from school staff, who could comment on their perspective of differences between current groups of children and similar aged children’s behaviours before the pandemic, also added nuance to findings. Many parents who do not have older children are unable to make such comparisons. The study was not without limitations, however. Participants tended to be white-British, active, and from higher socio-economic groups. Although we determined that the information power of the data collected was adequate for meeting the aims of this study and additional steps were taken to recruit participants from more diverse backgrounds, such as targeted recruitment and purposive sampling of children, we were unable to explore socio-economic issues in-depth. Future research exploring these issues among more diverse groups is therefore needed.

## Conclusions

The COVID-19 pandemic has influenced children’s physical activity. This qualitative study suggests that the physical activity patterns of children in the UK have changed a year following the lockdowns, with the continuation of sedentary lockdown habits within the home and the transition towards more organised physical activities, such as active clubs. Despite activity levels returning to pre-pandemic levels on average, girls and children from lower socio-economic groups may be at risk of decreased physical activity, with pre-pandemic issues being exacerbated during the pandemic.

## Supplementary Information

**Additional file 1.** Interview and focus group topic guides.

## Data Availability

As the Active-6 project is still ongoing, data are not currently available. Data will be made available in the University of Bristol’s data repository at the end of the project (https://data.bris.ac.uk/data/).

## Abbreviations

MVPA: Moderate to Vigorous Physical Activity
PE: Physical Education
TV: Television
