# Supplementary material for: The new normal for children’s physical activity and screen viewing: a multi-perspective qualitative analysis of behaviours a year after the COVID-19 lockdowns in the UK
Source: BMC Public Health. 2023 Jul 27;23:1432. doi: 10.1186/s12889-023-16021-y (PMC10373375; doi:10.1186/s12889-023-16021-y)
Supplement: Supplementary file 1 — Additional file 1. Interview and focus group topic guides. [file 12889_2023_16021_MOESM1_ESM.docx]

**The new normal for children's physical activity and screen viewing: A multi-perspective qualitative analysis of behaviours a year after the COVID-19 lockdowns in the UK**

**Authors:** Robert Walker, Danielle House, Ruth Salway, Lydia Emm-Collison, Lara E Hollander, Kate Sansum, Katie Breheny, Sarah Churchward, Joanna G Williams, Frank de Vocht, William Hollingworth, Charlie Foster, and Russell Jago.

**Supplementary File: Interview and focus group topic guides**

This file includes the guides for: A) Parent interviews; B) School staff interviews; C) Child focus groups.

1. **Parent interview guide**

**Introduction**

Thank you for agreeing to take part in this interview, your views and opinions are really important to us. In the interview today, I would like to talk about three main points:

- Whether there are any differences in your and your child’s pre-pandemic and recent physical activity patterns
- Factors you feel have influenced any changes in physical activity patterns
- Whether you think there is anything that can be done to help support you and your child’s physical activity

We are really interested in your honest opinions, we are not here to judge you, and we do not want you to feel like you should answer any of the questions in a certain way. There are no right or wrong answers, and as much detail you can give on the topics as possible is really appreciated.

*Confirm participant has read the information sheet/consent form and are still happy to participate. Gain verbal consent if participant has not consented during the Active-6 sign up process. If the participant is happy to proceed, the recording will start.*

**Changes in activity patterns**

***Parents***

1. Do you have any favourite physical activities? Why are they your favourite?
2. How would you describe your activity levels before the first lockdown (pre-March 2020)
   1. Did you often use active modes of transport?
   2. Were you part of any active clubs or have any active hobbies?
   3. Are you active around the house?
   4. If you are employed, is your work active?
3. To what extent do you feel that your current activity levels are different to pre-pandemic levels?
4. Can you describe the key factors that have influenced any changes to your activity patterns?

Prompts:

1. Priorities
2. Motivation
3. Discovering new types of activity in lockdown
4. COVID fears and worries
5. *Changes to family physical activity patterns*
6. In the first part of the Active-6 project, we found that, generally speaking, during the last half of 2021 parents were ***similarly*** active than a similar sample of Year 6 parents from 2018 (pre-pandemic). Does this finding match your experience, either personal or of those around you? Why do you think this may or may not be the case?
7. To what extent do you feel that any changes to your activity patterns are likely to last long-term or change in the future?
   1. Did your activity levels stay consistent throughout the pandemic? Or did you make an effort to increase/return to similar levels following the easing of restriction?

***Year 6 Child***

1. Does your Year 6 child have any favourite physical activities? Why do you think it’s their favourite?
2. How active was your child before the first lockdown (pre-March 2020)?
   1. Did your child use active modes of transport to school?
   2. Did they participate in any active clubs or hobbies?
   3. Does your child do any types of active play?
3. To what extent do you feel that your Year 6 child’s current activity levels are different to pre-pandemic levels?
4. Can you describe the key factors that have influenced any changes to your child’s activity patterns?

Prompts:

1. Active club provision/interest
2. Motivation
3. Confidence/self-efficacy
4. Changes to active play outside of school
5. Changes to child’s hobbies/interests
6. Conversely to what we found with parents, we observed that, generally speaking, during the last half of 2021 Year 6 children were ***less*** active than a similar sample of Year 6 children from 2018. Does this finding match your experience, either personal or of those around you? Why do you think this may or may not be the case?
7. To what extent do you feel that any changes to their activity patterns are likely to last long-term or change into the future?

***Screen time/electronic device use***

1. How would you describe your child’s current screen time/device use?
2. Has the way they use devices changed at all?
3. Do you feel that screen time/device use impacts physical activity levels?

Prompts:

- Have you ever changed the amount of leisure screen time your child is allowed because of increased online school work?
- How do you view TV vs phones/tablets/games consoles? Do you consider them the same?

***Solution focused ideas***

1. Are there any barriers that are ***currently*** preventing you/your child from being more active? How and who could support you to remove these barriers?
2. Is anything needed to help you and/or your child engage in more:
   1. Active travel (*i.e.* walking to school)
   2. Active clubs at school (*i.e.* school sports teams)
   3. Active clubs outside of school (*i.e.* community sports teams, scouts/brownies)
   4. Active play outside of school (*i.e.* playing football with friends, cycling, tag etc.)
   5. Family physical activities (*i.e.* family walks, family cycling, family swimming)

**Closing statement**

- Is there anything else you’d like to tell us about the things we talked about today?
- Do you have any questions for me?
- We appreciate you sharing your thoughts and opinions with us!

1. **School contact interview guide**

**Introduction**

Thank you for agreeing to take part in this interview, your views and opinions are really important to us. In the interview today, I would like to talk about three main points:

- Current physical activity patterns among Year 6s
- Whether there are any lasting changes since the pandemic within the school, and the extent to which you feel these impact physical activity among Year 6 children
- What kind of support could be provided to schools to help improve physical activity provision

We are really interested in your honest opinions, we are not here to judge you, and we do not want you to feel like you should answer any of the questions in a certain way. There are no right or wrong answers, and as much detail you can give on the topics as possible is really appreciated.

*Confirm participant has read the information sheet/consent form and are still happy to participate. Gain verbal consent if participant has not completed and returned their school contact consent form. If the participant has consented and is happy to proceed, the recording will start.*

**Child physical activity levels in school**

1. How active would you describe the ***current*** activity levels/patterns of the Year 6 pupils?
   1. How active are playtimes, generally?
   2. What is participation in active after-school clubs like? An active after-school club is a club at your school that is all about playing a sport or being active.
   3. How frequently do pupils use active modes of travel to get to and from the school? (*e.g.* walking, cycling, scooting)
   4. Are these physical activity patterns/levels different than before the pandemic?
2. Using the activity belt data we collected last year, we observed that, generally speaking, during the last half of 2021 Year 6 children were ***less*** active than a similar sample of Year 6 children from 2018.
   1. What do you think about this?
   2. Does this finding match your experience, either personal or of those around you?
   3. Why do you think this may or may not be the case?
3. What do you feel are the factors influencing any differences?

**Lasting structural changes within the school and their impact on physical activity**

1. After speaking with members of school staff last year, there was a lot of discussion surrounding differences/changes within the school due to the pandemic. Can you describe any differences that are still apparent within the school?
   1. Funding changes
   2. Restrictions on play equipment/facilities
   3. Bubbles/pods
   4. After school club provision
   5. Reductions in interschool sports/events
   6. Academic priorities
   7. Outdoor/active learning
2. To what extent do you feel that any changes are impacting physical activity among Year 6s?
3. To what extent do you anticipate any lasting changes within the school continuing in the future?

**Lasting changes within children**

1. Parents, children, and school staff last year spoke of changes within children, such as motivation or weight gain, that they had observed or experienced after the most recent reopening/easing of lockdown in April 2021. To what extent do you feel that any changes within children are still apparent at the moment?
   1. Emotional overwhelming and fatigue
   2. Social conflict/challenges during play/breaktimes
   3. Weight gain and loss of fitness
   4. Active club interest/participation
2. To what extent do you feel these changes are likely to continue in the future?

**Solution focused ideas**

One of our primary goals of the Active-6 project is to provide information to policy makers on what can be done to help support physical activity among children. As part of this, we are very interested in what you, and other members of staff, feel could be done to support physical activity at school. Your thoughts and opinions are really important to us. Please consider the following questions:

1. Do you feel there is anything needed to help Year 6 children engage in more:
   1. Active travel (*i.e.* walking to school)
   2. Active afterschool clubs (*i.e.* school sports teams)
   3. Active clubs outside of school (*i.e.* community sports teams, scouts/brownies)
   4. Active play during breaktimes or after school (*i.e.* playing football with friends, tag etc.)
2. Can you describe any challenges that you or other members of staff experience related to physical activity provision at school?
3. What do you and other members of staff feel could be done to support physical activity in school?
4. How does your school currently spend physical activity-related funding?
5. To what extent do you feel that funding for physical activity in school is sufficient?
6. If you are able to comment, to what extent do you feel the school’s use of funding (*i.e.* sport premium) is utilised effectively and appropriately?
7. If the school was provided with additional funding for physical activity and PE, how might you spend it?
8. How would you describe the school current afterschool club provision? What could be done to support/improve afterschool clubs?
9. To what extent do you feel the senior leadership team has an important role in physical activity in school?
10. To what extent do you feel teachers are supported in promoting physical activity?
11. To what extent do you agree/disagree that academic or other pressures negatively impact physical activity among Year 6s?
12. To what extent do you feel that teacher training is sufficient to deliver good physical activity/PE to children?
13. To what extent do you feel that the current PE curriculum is effective and engaging for Year 6 children? How do you think it could be improved?
14. To what extent do you physical activity and PE are prioritised at school?

**Closing statement**

- Is there anything else you’d like to tell us about the things we talked about today?
- Do you have any questions for me?
- We will be sharing the results with all participants once the study is complete.
- We appreciate you sharing your thoughts and opinions with us!

1. **Focus group plan and topic guide**

**Introduction**

Thank children for participating in the ACTIVE-6 project

- Introduce myself
- Explain why this project is important and what it means for them

***Activity 1: Making name tags***

- All children and focus group facilitators make name tags.

Give overview of focus group format:

- - We want to hear about your physical activity and how COVID-19 might have affected it
  - My role – I will be here to guide the discussion, but the discussion will mainly be among you all. Please feel free to ask each other questions and share your thoughts on what others say.
  - You are all the experts, so we really need your help!
  - Does everybody understand so far and does anyone have any questions?

***Activity 2: Hot potato***

Active game to discuss and conceptualise definition of physical activity. Ask the group to stand in a circle. Children then pass a ball (moon ball) or frisbee to the child on their left. When a child catches a ball they need to shout out a physical activity (*i.e.* football, walking to school). Repeating activities are not allowed. Child needs to put a hand behind their back if they cannot give a new answer. I will also participate to give examples of physical activities that might not be as obvious and help clarify the definition.

- Ethical procedures
  - If it is ok with you all, I will be **recording the conversation** to help me remember what we all talked about.
  - Show the microphone to the group
  - Someone will be writing this up word for word, so it is really important not to talk over each other so that the recording is clear
  - Your names and information will be completely removed from the write up, so no one can know who said what.
  - If you would like to leave at any time, that is perfectly fine, so please just let me know.
  - Does anyone have any questions for me?
  - Would anyone like to use the bathroom before we start?
- Is everyone happy to take part in this discussion?
- Is everyone happy for us to start? **If yes, begin the recording**

**Focus group discussion**

***Active travel***

1. How does everyone normally travel to and from school? **(keep brief)**
2. Have you always travelled to school and home that way, or has it changed?

Prompts:

- If they could, would anyone change the way they travel to and from school?
- Does anyone feel the way they travel to school now is different because of COVID?

**Physical activity during school time**

1. What is everyone’s favourite thing to do in PE? What do you like about it? **(keep brief)**

***Activity 3: Design your ideal PE lesson***

Working in groups of 2-3, the children will design their ideal PE session using whiteboards. These will then be used to facilitate discussions surrounding what might have changed and what could be done to promote physical activity. The groups will then take it in turns to describe their ideal lesson

Prompts:

- What do you think of your current PE lessons?
- How is your ideal PE lesson different or the same as your actual lesson?
- Have your PE lessons changed since COVID?

1. What kind of things do you all usually do at breaktimes? **(keep brief)**

***Activity 4: Design your ideal breaktime***

Working in groups of 2-3, the children will design their ideal breaktime using whiteboards. These will then be used to facilitate discussions surrounding what might have changed and what could be done to promote physical activity. The groups will then take it in turns to describe their ideal lesson

Prompts:

- What do you think of breaktimes at the moment?
- How is your ideal breaktime different or the same as your actual breaktime?
- Have your breaktimes changed since COVID?

**Physical activity outside of/after school**

***Active clubs***

Discuss definition of active clubs as “any organised group activity outside of school where you move your body, sometimes get out of breath, and heart beats a bit faster”, drawing on examples used during the hot potato activity – e.g. cubs/scouts too, not just sports.

1. What kinds of organised activities does everyone do after school? **(keep brief)**
2. When did everyone go back to their active club?
3. If at all, how have your clubs changed since COVID?
   1. Smaller groups
   2. Location
   3. Children/friends who attended
   4. Rules/restrictions
   5. Has anybody not gone back to the active club they did before COVID? Can you describe the reasons?
4. What kind of things does an active club need to have that would make you want to go?
   1. Friends attending
   2. What makes you feel comfortable/uncomfortable
5. Does anyone feel that the number of active clubs they do has changed?
   1. Has anybody recently started a new active club? What has that been like?
   2. Is anyone still not able to do the active club they would like to because of COVID, or another reason?
6. *Does anyone feel that there are certain clubs that are too expensive?*
   1. *Expensive kit*
   2. *Expensive travel*
   3. *Expensive cost*
7. *Can anyone describe a fun and cheap physical activity?*

***At home/screen time***

1. How does everyone spend their free time after school and at the weekends (outside of clubs)?

Prompts:

- 1. Who are you with?
  2. Spending time outdoors
  3. Playing outside alone or with others
  4. Play at home – crafts, baking, games etc
  5. Playing with friends or family online
  6. Watching TV or using electronic devices
  7. School work/study
  8. Other non-active activities

1. Who feels that the way they spend free time outside of school has changed since COVID?
   1. Would anybody like to play more in person more, but feel like they can’t? Why is this?
2. Does anyone feel their screen time or device use is different since COVID?
   1. Do you feel this impacts the amount of other activities that you do, such as playing outside?
   2. Can anyone describe their parent’s rules about screentime? What do you think about them?
   3. Do you think your parent’s rules about screen time have changed since the pandemic?
   4. Do you feel that using screens for schoolwork and screens for fun are the same or different?

**Exploring previous qualitative findings**

1. Did anyone feel more tired or find it difficult after COVID when everything like clubs and school reopened? How is this for you at the moment?
2. Did anyone find being around lots of people again after lockdown difficult, how are those feelings for you now?
3. Last year, I spoke to similar groups of year 6 children just before Christmas. These children spoke of not wanting to do as much physical activity as they did before COVID. They told me this was because they felt too tired because they had lost their fitness in lockdown and because of going back to school after lockdown and being surrounded by lots of other children.
4. What does everyone think of this?
5. Does anyone still feel this way, or did anyone feel this way at first but now feels better?
6. What made it feel better?
7. Would there be anything that could/ could have helped make it feel better?

**Closing statement and questions**

- Thank all the children and tell them they have been a big help with our research
- Overall, is there anything anyone would like to talk about or mention that we haven’t already discussed?
- Are there any questions?
- Say goodbye and end focus group
